# Designing *de novo* TIM barrels: insights into stabilization, diversification, and functionalization strategies

**DOI:** 10.1042/BST20253060

**Published:** 2026-02-11

**Authors:** Julian Beck, Sergio Romero-Romero

**Affiliations:** 1 Department of Biochemistry, University of Bayreuth, 95447 Bayreuth, Germany; 2 Department of Biochemistry and Structural Biology, Instituto de Fisiología Celular, Universidad Nacional Autónoma de México, 04510 Mexico City, Mexico

**Keywords:** (ɑ/β)8 barrel, AI-driven protein design, de novo protein design, physics-based design, protein scaffold, sequence-structure-function relationships

## Abstract

The triosephosphate isomerase (TIM)-barrel fold is one of the most versatile and ubiquitous protein folds in nature, hosting a wide variety of catalytic activities and functions while serving as a model system in protein biochemistry and engineering. This review explores its role as a key fold model in protein design, particularly in addressing challenges in stabilization and functionalization. We discuss historical and recent advances in *de novo* TIM barrel design from the landmark creation of sTIM11 to the development of the diversified variants, with a special focus on deepening our understanding of the determinants that modulate the sequence-structure-function relationships of this architecture. Also, we examine why the diversification of *de novo* TIM barrels toward functional diversification remains an open problem, given the absence of natural-like active site features. Current approaches have focused on incorporating structural extensions, modifying loops, and using cutting-edge AI-based strategies to create scaffolds with tailored characteristics. Despite significant advances, achieving enzymatically active *de novo* TIM barrels has been proven difficult, with only recent breakthroughs demonstrating functional activity. We discuss the limitations of stepwise design approaches and support integrated strategies that simultaneously optimize scaffold structure and active site shape, using both physics-based and AI-driven methods. By combining computational and experimental insights, we highlight the TIM barrel as a powerful template for custom enzyme design and as a model system to explore the intersection of protein biochemistry, biophysics, and design.

## *Designing the foundation:* why are TIM-barrels relevant to *de novo* protein design?

Proteins are biological machines, carrying out a myriad of functions in life. The ability to design proteins with tailored properties or functions offers great opportunities in biochemistry, biotechnology, or biomedicine, from new therapeutic options to the development of better industrial enzymes. Herein, the structural stabilization of proteins and the introduction of function represent two significant challenges within the context of protein design. While thermodynamic and kinetic stabilization allow proteins to exist stably under various conditions [1–6], functional integration involves improving some specific biochemical functions and amino acid geometries onto proteins of interest [7–10]. Tackling such issues requires a profound insight not only into the structure and dynamics of proteins but also a generally versatile scaffold amenable to successful design and engineering.

**Figure 1:**
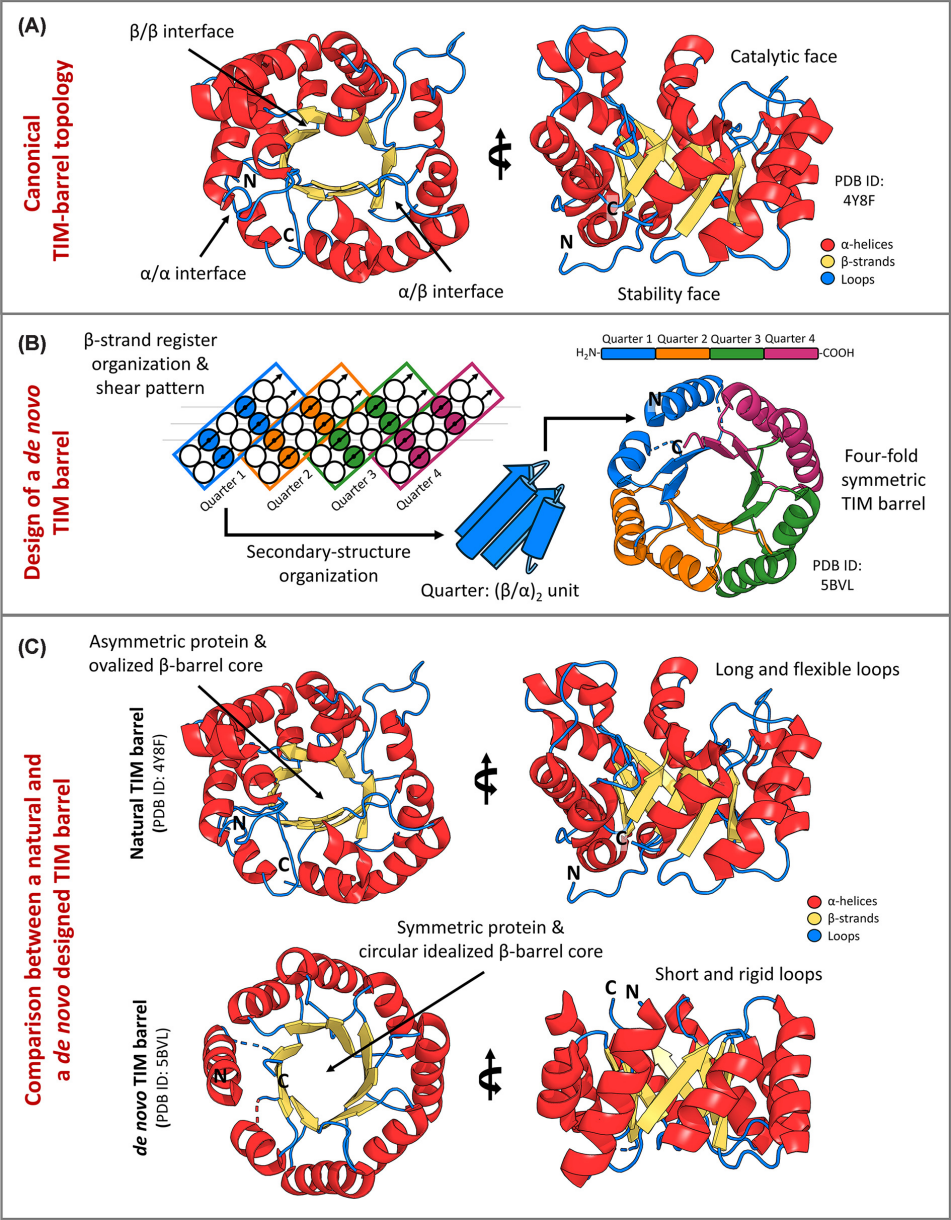
Structural features and design principles of TIM-barrel proteins (**A**) Cartoon representation of a canonical TIM-barrel fold (triosephosphate isomerase; PDB ID: 4Y8F), highlighting the overall (β/α)_8_ architecture. The β-barrel core provides a structurally stable scaffold, while the surrounding α-helices and the β–α loop regions form the so-called catalytic side, in contrast to the opposite face of the barrel, which primarily contributes to structural stability. (**B**) *De novo* design of sTIM11, illustrating the design strategy, its secondary-structure organization, and its four-fold symmetry. (**C**) Comparison of loop and core architectures between a natural TIM barrel (triosephosphate isomerase; PDB ID: 4Y8F) and a *de novo* design (sTIM11; PDB ID: 5BVL), emphasizing differences relevant to functional diversification.

One of the most ubiquitous and versatile topologies in nature, the triosephosphate isomerase (TIM)-barrel fold, has emerged as a key model system in protein design [11–16]. This fold was first identified in 1975-1976 through structural studies of TIM, giving rise to its eponymous name [17,18]. Composed of eight β-strands that alternate with eight α-helices, the TIM barrel possesses a partial symmetry and modular architecture [19–22]. This fold is remarkably versatile, supporting a wide range of catalytic functions with catalytic efficiencies that approach the diffusion limit, a property that has been linked to the high intrinsic stability of the TIM-barrel core (stability face) and to the spatial segregation between a conserved, stable scaffold and a highly adaptable active-site region located at the barrel termini (catalytic face) (Figure 1A). This organization allows for extensive variations in sequence and structure while preserving the core topology and overall fold integrity. Its ancient evolutionary origins further support its importance, being the TIM barrel a fold that has diversified across billions of years of evolutionary history, hosting multiple types of activities and functions [19,23–27].

Its size and complexity render the TIM barrel particularly appealing for protein design, a compromise between structural simplicity and architectural sophistication: its simplicity arises from a repetitive βα motif and a well-defined folding topology, while its sophistication lies in the precise spatial arrangement of these elements, which enables the formation of enclosed active-site pockets and supports a wide range of enzymatic activities. In contrast, simpler folds such as helical bundles or coiled-coil architectures, while highly tractable and already successfully engineered for complex functions [28–32], generally offer a more limited architectural framework for the emergence of diverse catalytic sites. These features make the TIM-barrel topology a well-defined framework in which protein design rules can be applied, including modular design strategies and principles derived from sequence–folding–structure relationships [33–39]. Besides, stability, dynamics, and modularity make it a suitable system for testing the effects of changes in sequence on protein stability and structure, thus offering an ideal architecture to study sequence–structure–function relationships.

In this mini-review, we discuss the role of *de novo* TIM barrels in protein design and engineering studies. We will also discuss how they serve as a model system in addressing fundamental questions in the field: stabilization via computational and experimental approaches, and diversification toward functionalization and tailor-enzyme design. By examining historic advances in context with more recent ones, we show how the unique properties of TIM barrels—ancient, versatile, designable, and amenable to investigations into sequence-structure-function relationships—have made them indispensable architectures for pushing the boundaries of protein science.

## *Designing for stability*: unraveling the stability landscape of *de novo* TIM barrels

**Figure 2:**
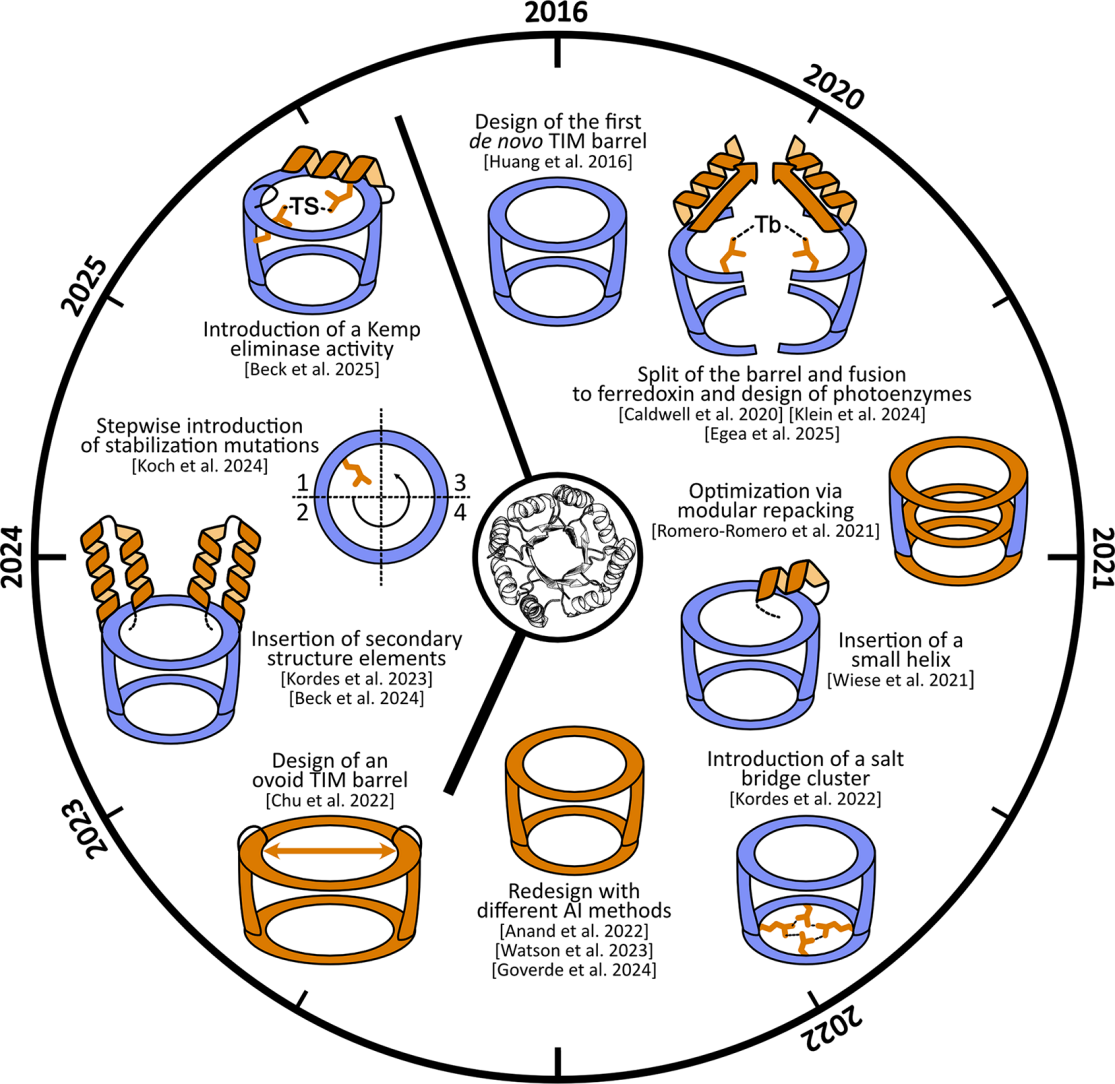
Schematic overview of all studes involving *de novo* TIM barrels The diagram begins with sTIM11, the first *de novo* TIM barrel, positioned at 12 o’clock, with its crystal structure as a white cartoon representation in the center (PDB: 5BVL). In the schematic, the TIM-barrel fold is simplified as a barrel. Blue indicates major preservation of the starting scaffold, while orange highlights the main objective or modification of each study. The timeline progresses clockwise, with the years on the outer edge marking the publication year of each reference or the first reference within a similar topic.

Protein stability is a crucial aspect to consider, as it directly affects functionality and applicability in biochemistry, biotechnology, and medicine. In this context, stability encompasses multiple, often interrelated properties, including thermodynamic and kinetic stability, resistance to proteases or aggregation, and robustness to environmental stresses such as temperature or chemical denaturants [2,3]. Importantly, protein stability and function are tightly coupled: insufficient thermodynamic or kinetic stability can limit functional expression and lifetime, whereas excessive rigidity may compromise the conformational dynamics required for catalysis or binding. Understanding and manipulating protein stability may not only provide key insights into the molecular determinants of the protein sequence-structure-function relationships but also increase our knowledge about the protein design rules [1,40,41]. In this framework, TIM barrels have been a model system to study how changes in the amino acid sequence influence protein stability and a long target of *de novo* protein design efforts. Table 1 and Figure 2 summarize the main studies and approaches in this field, as well as efforts toward diversification and functionalization, which are described in the next section.

**Table 1:** Summary of *de novo* designed TIM barrels to date and their design strategies

Protein/Family name	PDB IDs of related structures	Approach classification	Brief description and design strategy	Reference
**Aim: Stabilization**				
sTIM11	5BVL	Physics-based	The first structurally validated *de novo* TIM barrel, designed using Rosetta to build a 4-fold symmetric scaffold	[12]
sTIM11noCys	6YQY	Physics-based	Cysteine-free variant from sTIM11; this modification prevents disulfide bond formation, enhancing scaffold applicability	[42]
DeNovoTIMs	6Z2I (DeNovoTIM6), 6YQX (DeNovoTIM13)	Physics-based	A series of *de novo* TIM barrels with diverse thermal and conformational stabilities, designed using Rosetta with a modular approach to enhance hydrophobic core packing and improve sTIM11 stability	[42]
DeNovoTIM15	6WVS	Physics-based	Circularly permutated variant of DeNovoTIM13, designed using RosettaRemodel to revert the circular permutation of the original sTIM11	[83]
DeNovoTIMs-SB	7OSU and 7OT7 (sTIM11noCys-SB), 7OSV and 7OT8 (DeNovoTIM6-SB), 7P12 (DeNovoTIM13-SB)	Physics-based	DeNovoTIM variants designed using Rosetta based on natural TIM barrels, incorporating central salt bridge clusters to enhance structural stability and facilitate crystallization	[43]
DeNovoTIMs-quarters	No structures available	Physics-based	TIM barrel proteins with quarter-turn symmetry mutations, designed to explore the role of non-additive effects in protein stability	[44]
**Aim: Diversification and functionalization**				
TIM-FDs	6WXO (TFD-HE), 6WXP (TFD-EE), 6ZV9 (TFD-EE N6W)	Physics-based	Variant with a large internal cavity featuring a lanthanide-binding site for metal coordination, created by fusing two domains: a *de novo* designed dimeric ferredoxin fold and DeNovoTIM15	[83]
sTIM11_helix3	7A8S	Physics-based	Extension of the sTIM11noCys variant using Rosetta, incorporating a small helix at the top of the barrel for structural enhancement	[77]
OvoidTIM3	7UEK	Physics-based	A two-fold symmetrical design of an ovoid-shaped TIM barrel, with structural loops generated via RosettaRemodel	[74]
F-barrels	7MCC (F2C), 7SMJ (F2N), 7MCD (F15C)	AI-based	Variants with sequences derived from a learned potential using a deep neural network model	[72]
αTIMs (αTIM2 and αTIM2-2)	No structures available	Physics-based	Rationally designed coiled coils introduced into the top region of sTIM11noCys using RosettaRemodel	[78]
TIM_barrel_6	No structure available	AI-based	Non-idealized TIM barrel protein designed with RFdiffusion for structural and sequence variation	[65]
TBF_24	8OYS	AI-based	Redesign of TIM barrel proteins through an inversion of AlphaFold2 coupled with ProteinMPNN to mimic the topology of natural membrane proteins	[73]
HalluTIMs	8R8N (HalluTIM2-2), 8R8O (HalluTIM3-1)	AI-based	Structural extensions of DeNovoTIMs with multiple α-helical hairpins, designed via constrained hallucination	[80]
PhotoLanZymes + TFD variants	9QUC (TFD-EH), 9QUD (TFD-EH Cu^2+^), 9QUI (TFD-EH Ni^2+^), 9QUL (TFD-EH T87E Zn^2+^), 9QUO (TFD-EH T87E Co^2+^), 9QUP (TFD-EE MPNN)	Structure-based engineering	*De novo* designed photoenzymes (cerium-dependent enzyme) based on a previously designed TIM-barrel scaffold (TIM-FD), enabling C–C bond cleavage of 1,2-diols in aqueous solution via photoredox catalysis. Rational mutations modified metal-binding specificity and conformational behavior. AI-guided stabilization of the active conformation resulted in an improved enzyme.	[85,86]
KempTIMs	9QKX	Physics-based + AI-based	Enzymatically active *de novo* TIM barrel (Kemp eliminase). KempTIMs were designed using the CANVAS computational workflow, incorporating AI tools for structural extensions and the physic-based software Triad for transition state placement and pocket design.	[88]

In 2016, Huang and co-authors reported a four-fold symmetric *de novo* TIM-barrel protein [12], constituting an important landmark in this field so far, due to the historical difficulty of obtaining accurate and stable structures of this kind. This work used the Rosetta software to follow a bottom-up approach based on geometric and chemical principles. In addition, it provided design rules that included specific side chain-backbone hydrogen-bonding interactions that were critical for sustaining strand registers across repeat units (Figure 1B). By introducing a circular permutation that places the N-terminus at the beginning of an α-helix and repositions the chain termini into a loop region with minimal coupling to the core, they further stabilized their designs, albeit with a slight deviation from the canonical (β/α)_8_ topology, resulting in an (α/β)_8_ barrel instead. One of these circular permuted designs was sTIM11, an experimentally validated and thermostable protein of 184 residues that demonstrated proper secondary and tertiary structure element arrangements, as indicated in the Rosetta model. Importantly, sTIM11 exhibited an idealized structure with short loops and a circular core compatible with the intended TIM barrel topology (Figure 1C), despite having a sequence unrelated to natural TIM barrels. The successful design of sTIM11 as a circularly permuted TIM barrel demonstrates that the fold tolerates substantial topological rearrangements while preserving its structural integrity, highlighting its remarkable designability and flexibility as a protein scaffold. At the same time, natural TIM barrels exhibit substantial structural diversity while preserving the same core topology (often displaying a slightly ovalized β-barrel core, in contrast with the more circular geometry typically observed in *de novo* designs; Figure 1C), reflecting the effects of evolutionary optimization versus idealized design.

Using sTIM11 as a scaffold, Romero-Romero et al. designed a large family of *de novo* TIM barrels, DeNovoTIMs, using a computational fixed-backbone and modular approach with a focus on improving stability by enhancing hydrophobic packing based on the first validated *de novo* TIM barrel, sTIM11 [42]. DeNovoTIMs exhibited a wide range of both thermal and conformational stabilities, extending their applications in biochemistry, as will be further discussed in the next section. Furthermore, thermodynamic analyses suggested that large non-additive effects modulate the stability effects, pointing out that the stability of DeNovoTIMs is tunable by epistatic effects, i.e., interactions between mutations across different regions of the barrel.

In follow-up work, Kordes et al. tested the impact of introducing a salt bridge cluster on the structural and biophysical properties of three previously designed DeNovoTIMs [43]. While the salt-bridge variants (named DeNovoTIM-SBs) retained similar thermostabilities compared to the parental proteins, they showed differences in conformational stabilities at room temperature and different crystallization tendencies. Besides, the structural analyses showed that the geometries of the salt bridges across the proteins varied, directly influencing their stability and crystallization properties. This work underlined the complexity of the prediction of salt bridge interactions, suggesting that the design of such clusters in *de novo* proteins is an open field for further investigations.

Recently, Koch and colleagues studied the effects of the stepwise introduction of stabilizing mutations across the four TIM-barrel quarters on the thermal and conformational stability properties [44]. The study was built upon the foundation of earlier designs, particularly those of members of the DeNovoTIMs collection. They introduced mutations in a quarter-wise manner from DeNovoTIM0, the non-stabilized design, to DeNovoTIM6, a stabilized barrel, and observed non-linear and non-additive effects of stability, meaning that the number and location of mutations strongly influence overall protein stability. Accordingly, equivalent mutations in different structural positions of the protein also altered the stability, emphasizing the importance of effective packing and hydrophobic interactions to be achieved in such a closed architecture as the TIM-barrel fold. Results showed the complexity of modulating protein stability by sequence changes and gave valuable insights into protein design and engineering.

Overall, this stability landscape navigation by modifying the protein sequence has not only increased our knowledge about how stability can be fine-tuned by protein design but also expanded the repertoire of synthetic proteins. It laid the foundational principles that could enable the design of other complex protein architectures and, therefore, opened up possibilities for customized enzyme design.

## *Designing for functionality:* diversification and optimization strategies for tailored purposes

The design of the first *de novo* TIM barrel in 2016 [12] raised hopes that tailor-made enzymes were within reach. However, despite the passing time and recent advances in protein design based on the introduced AI tools, progress toward robust functional integration has not yet fully met these high expectations. Importantly, the relatively small number of recent functionalized TIM barrels or other *de novo* topologies reflects both the intrinsic difficulty of engineering active sites within a highly idealized scaffold and the long experimental timelines required for validation. A comparison of *de novo* TIM barrels to natural ones sheds light on the underlying challenges of functionalization. Natural TIM barrels form pockets using extended loops, secondary structure elements, or even additional domains, often anchoring critical catalytic residues within these extensions [11,19,27]. These structural extensions, particularly the extended loops, also provide dynamic motions on multiple time scales, enabling several of the exceptional functional properties observed in enzymatic TIM barrels [36,45–53]. In contrast, *de novo* TIM barrels lack these features; their highly idealized architectures do not readily support any pocket formation nor functional dynamics. Prior efforts exploring loop design, insertion, and grafting, as discussed below, highlight both the potential and the difficulties of introducing functional dynamics into otherwise idealized TIM-barrel scaffolds. More broadly, these difficulties reflect a general and persistent enduring limitation in protein design: the reliable design of loop regions that couple structure, dynamics, and function.

In this broader context, loop regions play a central role in protein function by mediating molecular recognition, catalysis, and conformational dynamics, yet they remain among the most challenging elements to design accurately. Unlike regular secondary structure elements, loops exhibit substantial conformational heterogeneity and are often sensitive to subtle sequence and environmental changes. Although recent advances in protein engineering and design, including loop grafting and insertion [54–56], deep learning-based structure prediction [57–63], and generative sequence & structure design methods [64–67], have significantly improved our ability to model loop conformations, reliable control over loop flexibility, dynamics, and functional positioning remains limited [68–70]. Notably, recent strategies have shown that loops can be reliably structured when buttressed by extensive stabilizing interactions within specialized scaffolds [71], enabling functional binding sites, but such approaches are not yet broadly transferable across protein architectures, such as TIM barrels. As a result, loop regions continue to represent a major bottleneck for translating structural designs into robust functional proteins.

These challenges are particularly pronounced in the context of TIM-barrel enzymes, where loop regions frequently define the active site architecture and govern access, specificity, and catalytic efficiency. In natural TIM barrels, extended and dynamic loops often cooperate with secondary-structure embellishments or auxiliary domains to create well-defined pockets and enable functionally relevant motions across multiple time scales. In contrast, most *de novo* TIM barrels adopt highly idealized architectures with short, rigid loops that prioritize stability over functional flexibility (Figure 1C). While this design choice has been instrumental in achieving robust folding and expression, it also limits pocket formation and the emergence of catalytically competent dynamics, underscoring loop design as a critical frontier for future TIM-barrel functionalization. These features help explain why TIM barrels uniquely combine high thermodynamic stability with functional plasticity, a property that has made them one of the most successful and recurrent enzymatic folds in nature.

As both a key model system in protein design and a particularly demanding design target, the TIM-barrel fold was used by multiple groups to validate their design pipelines. Anand and colleagues tested the capability of a deep neural network model for sequence design, achieving multiple well-behaved proteins (hereafter referred to as F-barrels for simplicity) and adding several *de novo* TIM barrels to the existing repertoire [72]. In another work, Goverde and colleagues developed a deep learning pipeline to design complex folds and soluble analogs of integral membrane proteins [73]. This pipeline resulted in new *de novo* TIM barrels with diversified sequences even breaking the established four-fold symmetry. Additional *de novo* TIM barrels were generated by Watson and colleagues to demonstrate that their generative diffusion algorithm (RFdiffusion) can be conditioned towards specific folds [65]. The proteins generated exhibit the expected circular dichroism and a high thermostability, but a detailed structural characterization was not performed. Despite another focus in all these studies, they diversified *de novo* TIM barrels to the existing repertoire either on the structural or sequence level.

To achieve a different structural starting point compared to the circular sTIM11 and its descendants, Chu *et al.* designed a *de novo* TIM barrel with an ovoid shape [74]. This ovoid curvature aimed to accommodate residue combinations and networks within the core that might not be feasible within a circular TIM barrel. For the design of an ovoid TIM barrel, they constructed a blueprint for an ovoid topology of the inner β-barrel guided by a two-fold symmetry, varying the curvature and structural parameters such as the shear number. An autoregressive sampling strategy was used to sequentially build each secondary structure element to achieve optimal α-helix and loop lengths to support the ovoid curvature, and sequence design was performed via an iterative enrichment protocol with Rosetta [75,76]. The resulting ovoid TIM barrels showcased high thermodynamic stability, and a solved crystal structure confirmed a high similarity to the computational model and the intended ovoid geometry. Notably, additional features favorable for downstream introduction of function include βα-loops, which are free of any critical hydrogen bonding, and a high tolerance of the inner core to polar or charged residues eventually necessary for function. While these features make the ovoid TIM barrels a good alternative starting point, some critical limitations remain, like the absence of pockets, extended loops, or structural extensions to accommodate functional residues, rendering subsequent function integration a demanding task.

To move towards functionalization with another strategy, multiple secondary structure elements have been added on top of sTIM11 and its descendants, aiming to achieve additional surface area and potential catalytic pockets. For instance, Wiese *et al.* incorporated a small α-helix into the scaffold, a common motif in natural TIM barrels often involved in phosphate binding [77]. Their approach involved Rosetta *ab initio* structure prediction of the intended α-helix, its attachment to the scaffold, and Rosetta optimization. However, the crystal structure showed significant deviation from the intended design, with the desired α-helix adopting a 3_10_ configuration. This discrepancy, combined with the small size of the added motif, limited the potential for further engineering of the motif.

In another study, Kordes *et al.* introduced substantially larger secondary structure elements (helix-loop-helix motifs) to significantly expand the surface area and establish a pocket above the barrel, generating so-called αTIMs [78]. Their workflow for inserting a single helix-loop-helix motif involved rational design, Rosetta *ab initio* structure prediction, attachment to the scaffold, and optimization of the motif and its transition region into the barrel using Rosetta. As initial experiments indicated the successful extension, the scaffold's four-fold symmetry was leveraged to duplicate the motif in another half of the barrel, enhancing the surface area further and potentially forming a pocket between the extensions. While no experimental structure was obtained, AlphaFold2 predictions supported the Rosetta models, and PUResNET [79] predicted the formation of a pocket above the inner β-barrel, achieving the design objectives.

Notably, both extended TIM barrels relied on Rosetta *ab initio* structure prediction rather than the now-established AlphaFold2 [60]. This is easily explained by the fact that both studies began long before AlphaFold2's release, underscoring the lengthy timeline of efforts to diversify *de novo* TIM barrels. In contrast, a more recent study by Beck *et al.* used AlphaFold2 alongside other AI tools for sequence and structure diversification [80]. Unlike earlier approaches based on rational design, their method relied on constrained hallucination, which is theoretically capable of generating diverse structural elements [81,82]. Despite this potential, Beck *et al.* obtained exclusively helical extensions closely resembling the helix-loop-helix motif previously introduced by Kordes *et al.* A notable advancement was the successful addition of an extra extension, increasing surface area and enhancing potential pocket interactions. However, crystal structures and SAXS data indicated substantial flexibility in the extensions, rendering functionalization of the predicted pocket particularly difficult.

One additional downside of these stepwise introductions of pockets for downstream introduction of function is that they are introduced as generalizable pockets without considering a certain enzyme or binding activity. This might be advantageous initially, but introducing function, specifically enzymatic activity, requires exact control over the geometry of the catalytic residues and their surroundings. The chances that a premade pocket can accommodate the desired reaction and catalyze it with desirable efficiency are unlikely, making the strategy of stepwise diversification towards functionality uncertain.

Despite these limitations, Caldwell *et al.* demonstrated an outstanding example through the successful stepwise introduction of a binding functionality into a highly modified *de novo* TIM barrel [83]. The initial step involved further optimization and reversal of the circular permutation in the already optimized DeNovoTIMs, resulting in DeNovoTIM15 with an additional βα-loop compared to the existing DeNovoTIMs. Additionally, they utilized the barrel's four-fold symmetry by splitting it into two halves, achieving a complete TIM barrel through homodimerization. By fusing a monomer of a *de novo*-designed ferredoxin [84], they generated a highly stable homodimeric fusion protein, TIM-FD (TFD), which forms a substantial internal cavity above the barrel, between the domains. To functionalize the designed cavity, they introduced two glutamates to build up a metal coordination site for large trivalent cations (TFD-EE variant). They showcased lanthanide binding with a high affinity via spectroscopic measurements and a high-resolution crystal structure. With the binding of lanthanides and the availability of designable residues in the cavity for substrate recognition, the protein shows potential for downstream enzyme design. Nonetheless, it still faces the drawbacks of a premade pocket, which requires further modifications to achieve the necessary customizability for tailor-made enzymes.

Building upon this design, Klein et al. recently repurposed the TFD scaffold to create a *de novo* photoenzyme for lanthanide-mediated photoredox catalysis (PhotoLanZyme) [85]. The design incorporated a tetraglutamate Ce^3+^ binding site in the internal cavity, enabling light-triggered ligand-to-metal charge transfer that initiates selective C–C bond cleavage in 1,2-diols. To improve metal specificity and reduce unspecific terbium binding to the protein surface, the authors identified exposed acidic residues, mutating six aspartates and two glutamates to neutral residues, yielding PhotoLanZyme version 1.4. Additionally, the engineered enzyme retained function when expressed on the surface of *E. coli*, enabling photobiocatalysis in whole cells. This work exemplifies how a well-characterized, modular *de novo* scaffold can be progressively adapted toward increasingly complex and abiological catalytic functions.

In a follow-up study, Egea and colleagues aimed to alter the metal specificity toward divalent first-row transition metal ions by introducing the mutation E154H into each monomer of the scaffold (TFD-EH) (Egea et al. 2025) [86]. The introduced mutation enabled binding of divalent metals, as demonstrated by crystal structures of complexes with Cu^2+^ and Ni^2+^, in which even two ions were bound. To further stabilize a dinuclear binding mode, an additional coordinating residue (T87E) was introduced. Crystal structures with Zn^2+^ and Co^2+^ demonstrated the participation of this residue and revealed a different binding coordination compared to before. As the TFD scaffold contains tri-glycine linkers, the authors also investigated the flexibility of their variants, revealing that the TFD scaffold is not rigid and that its flexible linkers enable dynamic motions. Furthermore, this analysis showed that TFD-EE adopts a conformational equilibrium between two states. To shift the equilibrium toward the active state, the scaffold was optimized using ProteinMPNN. The resulting protein, TFD-EE MPNN, formed only a single dimeric species and exhibited a 10-fold increase in catalytic efficiency compared to the original scaffold. This study highlights the possible plasticity of *de novo* scaffolds and demonstrates the potential of multi-domain architectures with flexible linkers to enable conformational dynamics in hyperstable *de novo* proteins

To harness the full enzymatic potential of *de novo* TIM barrels, a paradigm shift in their functionalization is necessary. Achieving the required accuracy for a desired enzymatic reaction with sufficient efficiency through a stepwise addition of extensions and functional sites is exceedingly difficult [87]. Only recently Beck and colleagues achieved an enzymatic active *de novo* TIM barrel based on the simultaneous introduction of an extension to form an active site for a specific reaction [88]. With their so-called CANVAS approach (Customizing Amino-acid Networks for Virtual Active-site Scaffolding), which includes physics-based software like Triad [89] and AI-based tools like RFdiffusion [65], they introduced a Kemp eliminase activity—a benchmark reaction in computational enzyme design for carbon-based proton transfer—into a *de novo* TIM barrel with the canonical (β/α)_8_ topology, obtained through a reversal circular permutation of DeNovoTIM6-SB.

One active variant showcased a comparable efficiency to traditionally designed Kemp eliminases with transition state placements in existing scaffolds [90], despite the additional challenge of building up structural extensions for the active site. Nonetheless, in comparison to recently achieved catalytic efficiencies in *de novo* enzyme design with natural and entirely newly created scaffolds [91–95], the achieved efficiency remains lower and does not yet reach the levels that represent the desired outcome of *de novo* enzyme design or the full potential of the TIM-barrel fold. Nonetheless, with these recent advances in enzyme design, alongside progress in general protein design and the emergence of AI-based tools [66,96–101], we are optimistic that increasingly complex and efficient enzymatic TIM barrels and the exploration of their full enzymatic potential are on the horizon.

## *Designing beyond*: concluding perspectives and challenges in the field

The TIM-barrel architecture represents an optimal topology to apply protein design rules and increase our understanding of protein structure and function while unlocking new applications in biochemistry and synthetic biology. As discussed in this review, the successful design of TIM-barrel proteins highlights the importance of coupling computational techniques with experimental validation. Recent advances in computational protein design, particularly through the application of physics-based methods and deep learning algorithms, have led to the creation of novel TIM-barrel members with unprecedented stability, sequence diversity and, recently, catalytic activity. At the same time, the relative difficulty of extending the functional repertoire of *de novo* TIM barrels, especially when compared to simpler, more readily functionalizable scaffolds, raises important questions about their continued relevance as design targets.

Despite these challenges, pursuing the design of more advanced *de novo* TIM barrels remains worthwhile. While other scaffolds, such as *de novo* helical bundles, have proven more readily functionalizable, TIM barrels address a distinct and complementary challenge in protein design. Natural TIM barrels exhibit an exceptional combination of high stability, finely tuned dynamics, and remarkable enzymatic efficiency, making them one of the most successful enzymatic architectures in nature. Understanding how these properties arise within a complex yet highly ordered topology may enable the formulation of transferable design principles, facilitating both the design of improved *de novo* variants and the extension of these insights to other protein folds.

One of the key difficulties is extending the functional profile of the designed and engineered proteins, as naturally occurring TIM barrels can bind complex ligands or catalyze complex reactions. It calls for tight control of binding sites and active site geometries to introduce novel functions or cofactors. Although expanding the TIM barrel topology by adding other functional domains or motifs can lead to novel scaffolds with improved traits, this requires a better understanding of how different structural elements can be properly fused without loss of stability or function. In particular, the design and control of flexible loops, which provide the necessary dynamics to approach the enzymatic efficiency of natural TIM barrels, remain major obstacles, as current design algorithms still preferentially generate rigid extensions. Ultimately, it should even be considered to shift from fusing existing TIM barrels to designing an entirely new TIM barrel in the context of the desired function, as this could lead to a superior interplay between extensions and core, enabling a broader range of possible topologies for extensions.

In this regard, recent advances in generative protein design are beginning to reshape how complex folds such as TIM barrels can be approached. Deep learning–based methods, including structure- and sequence-level generative models [65,73,102], enable the simultaneous optimization of global scaffold architecture and local functional features, such as active-site geometry and residue composition, enabling greater control over shape, size, and overall complexity. These developments are particularly relevant in the context of enzyme design, where traditional approaches often yield low-activity catalysts that require extensive experimental optimization and high-throughput screening. Rather than treating the TIM barrel as a fixed scaffold to be functionalized in a stepwise manner, these approaches open the possibility of co-designing the fold and its functional elements from the outset. Recent hybrid strategies that combine generative models with atomistic active-site scaffolding have demonstrated near-atomic precision and catalytic activities approaching those obtained through directed evolution, highlighting the potential of integrated, function-first design paradigms [93]. As these methods continue to mature, they may help overcome some of the historical limitations associated with TIM-barrel functionalization and reframe this fold as a viable target for integrated, function-first design strategies.

Furthermore, protein dynamics is crucial for enzymatic function, yet most design approaches target static structures. Capturing and designing conformational flexibility remains a significant task since most traditional structure prediction algorithms predict single, rigid conformations. To design highly functional TIM-barrel enzymes, it is crucial to validate and apply new methods that incorporate dynamics explicitly, either through molecular dynamics simulations or AI-based approaches.

Having achieved all these insights through work on the TIM-barrel fold, it is also worth exploring how such design principles could be applied or extended to other protein topologies. While TIM barrels provide a robust and adaptable scaffold, certain catalytic activities or binding specificities might be better suited by alternative architectures (e.g., α/β-hydrolases, Rossmann folds, or entirely novel *de novo* scaffolds), which also serve as valuable benchmarks for assessing the strengths and limitations of TIM barrels. By applying computational and experimental strategies developed in the context of TIM-barrel design, it is now feasible to generate entirely new protein folds specific to functional requirements. This expansion beyond TIM barrels could lead to new enzymatic activities and increase the number of *de novo*-designed proteins available for new applications.

Finally, as has been discussed, a promising direction for the field is the integration of physics-based and AI-driven approaches. While physics-based design provides mechanistic insight into the design process and energy calculation, AI methods offer effective sequence and structure optimization. Assessing the performance of these combined approaches along with addressing the challenges in the field might lead to the generation of more robust and efficient proteins, reducing costs and time for the computational and experimental characterization, and allowing the design of more complex artificial proteins with tailored functionalities.

## Perspectives

- **Highlight the importance of the field:** This manuscript highlights the TIM-barrel fold as a key model in protein design, emphasizing its versatility and challenges in functionalization. This review was undertaken to explore recent progress in TIM barrel design, identify key limitations in enabling catalytic functions, and discuss emerging strategies, particularly AI-driven approaches, to overcome these obstacles and expand the utility of TIM barrels in protein engineering.
- **Summary of the current thinking:** The review outlines advances in *de novo* TIM barrel design, highlighting current limitations in functionalization and recent AI-driven strategies. It emphasizes the need for integrated approaches to optimize both scaffold stability and active site formation, paving the way for custom enzyme development.
- **Comment on future directions:** While significant advances have been made in *de novo* TIM barrel design, achieving enzymatic activity remains difficult due to the lack of natural-like active sites. Current strategies involve structural modifications and AI-driven approaches to tailor scaffolds for specific functions. These insights have important implications for human health and disease, as engineered TIM barrels could lead to novel enzymes for drug development, enzyme replacement therapies, diagnostics, and biocatalysis. By integrating computational and experimental methods, TIM barrels serve as a powerful template for custom enzyme design with potential biomedical and biotechnological applications.

## Data Availability

All data are included in the main manuscript.

## Open Access

Open access for this article was enabled by the participation of Universidad Nacional Autonoma de Mexico in an all-inclusive Read & Publish agreement with Portland Press and the Biochemical Society.

## Abbreviations

TIM: triosephosphate isomerase

## References

[1] Goldenzweig A. and Fleishman S.J. (2018) Principles of protein stability and their application in computational design. Annu. Rev. Biochem. 87, 105–129 10.1146/annurev-biochem-062917-012102 29401000

[2] Romero Romero S., Fernández Velasco D.A. and Costas M. (2018) Estabilidad termodinámica de proteínas. Educ. Quim. 29, 3 10.22201/fq.18708404e.2018.3.64699

[3] Sanchez-Ruiz J.M. (2010) Protein kinetic stability. Biophys. Chem. 148, 1–15 10.1016/j.bpc.2010.02.004 20199841

[4] Pace C.N. and Grimsley G.R. (2001) Protein stability. Encyclopedia of Life Sciences, Wiley, 2001 [cited 2025 Feb 2] 10.1038/npg.els.0003002

[5] Fersht A.R., Jackson S.E. and Serrano L. (1993) Protein stability: experimental data from protein engineering. Philos. Trans. R. Soc. Lond. Ser. Phys. Eng. Sci. 345, 141–151 10.1098/rsta.1993.0125

[6] DeGrado W.F., Wasserman Z.R. and Lear J.D. (1989) Protein design, a minimalist approach. Science 243, 622–628 10.1126/science.2464850 2464850

[7] Pan X. and Kortemme T. (2021) Recent advances in de novo protein design: principles, methods, and applications. J. Biol. Chem. 296, 100558 10.1016/j.jbc.2021.100558 33744284 PMC8065224

[8] Khakzad H., Igashov I., Schneuing A., Goverde C., Bronstein M. and Correia B. (2023) A new age in protein design empowered by deep learning. Cell Syst. 14, 925–939 10.1016/j.cels.2023.10.006 37972559

[9] Notin P., Rollins N., Gal Y., Sander C. and Marks D. (2024) Machine learning for functional protein design. Nat. Biotechnol. 42, 216–228 10.1038/s41587-024-02127-0 38361074 PMC13159571

[10] Lechner H., Ferruz N. and Höcker B. (2018) Strategies for designing non-natural enzymes and binders. Curr. Opin. Chem. Biol. 47, 67–76 10.1016/j.cbpa.2018.07.022 30248579

[11] Romero-Romero S., Kordes S., Michel F. and Höcker B. (2021) Evolution, folding, and design of TIM barrels and related proteins. Curr. Opin. Struct. Biol. 68, 94–104 10.1016/j.sbi.2020.12.007 33453500 PMC8250049

[12] Huang P.-S., Feldmeier K., Parmeggiani F., Fernandez Velasco D.A., Höcker B. and Baker D. (2016) De novo design of a four-fold symmetric TIM-barrel protein with atomic-level accuracy. Nat. Chem. Biol. 12, 29–34 10.1038/nchembio.1966 26595462 PMC4684731

[13] Offredi F., Dubail F., Kischel P., Sarinski K., Stern A.S., Van De Weerdt C. et al. (2003) De novo backbone and sequence design of an idealized α/β-barrel protein: evidence of stable tertiary structure. J. Mol. Biol. 325, 163–174 10.1016/S0022-2836(02)01206-8 12473459

[14] Figueroa M., Oliveira N., Lejeune A., Kaufmann K.W., Dorr B.M., Matagne A. et al. (2013) Octarellin VI: using rosetta to design a putative artificial (β/α)8 protein. PloS ONE 8, e71858 10.1371/journal.pone.0071858 23977165 PMC3747059

[15] Tanaka T., Hayashi M., Kimura H., Oobatake M. and Nakamura H. (1994) De novo design and creation of a stable artificial protein. Biophys. Chem. 50, 47–61 10.1016/0301-4622(94)85019-4 8011940

[16] Beauregard M., Goraj K., Goffin V., Heremans K., Goormaghtigh E., Ruysschaert J.-M. et al. (1991) Spectroscopic investigation of structure in octarellin (a *de novo* protein designed to adopt the α/β-barred packing). Protein Eng. Des. Sel. 4, 745–749 10.1093/protein/4.7.745 1798699

[17] Banner D.W., Bloomer A.C., Petsko G.A., Phillips D.C., Pogson C.I., Wilson I.A. et al. (1975) Structure of chicken muscle triose phosphate isomerase determined crystallographically at 2.5Å resolution: using amino acid sequence data. Nature 255, 609–614 10.1038/255609a0 1134550

[18] Banner D.W., Bloomer A.C., Petsko G.A., Phillips D.C. and Wilson I.A. (1976) Atomic coordinates for triose phosphate isomerase from chicken muscle. Biochem. Biophys. Res. Commun. 72, 146–155 10.1016/0006-291X(76)90972-4 985462

[19] Nagano N., Orengo C.A. and Thornton J.M. (2002) One fold with many functions: the evolutionary relationships between TIM barrel families based on their sequences, structures and functions. J. Mol. Biol. 321, 741–765 10.1016/S0022-2836(02)00649-6 12206759

[20] Wierenga R.K. (2001) The TIM‐barrel fold: a versatile framework for efficient enzymes. FEBS Lett. 492, 193–198 10.1016/S0014-5793(01)02236-0 11257493

[21] Höcker B., Claren J. and Sterner R. (2004) Mimicking enzyme evolution by generating new (βα)_8_-barrels from (βα)_4_-half-barrels. Proc. Natl. Acad. Sci. 101, 16448–16453 10.1073/pnas.0405832101 15539462 PMC534502

[22] Lang D., Thoma R., Henn-Sax M., Sterner R. and Wilmanns M. (2000) Structural evidence for evolution of the β/α barrel scaffold by gene duplication and fusion. Science 289, 1546–1550 10.1126/science.289.5484.1546 10968789

[23] Copley R.R. and Bork P. (2000) Homology among (βα) 8 barrels: implications for the evolution of metabolic pathways 1 1Edited by G. Von Heijne. J. Mol. Biol. 303, 627–641 10.1006/jmbi.2000.4152 11054297

[24] Henn-Sax M., Höcker B., Wilmanns M. and Sterner R. (2001) Divergent evolution of (betaalpha)8-barrel enzymes Biol. Chem. 382, 1315–1320 10.1515/BC.2001.163 11688714

[25] Anantharaman V., Aravind L. and Koonin E.V. (2003) Emergence of diverse biochemical activities in evolutionarily conserved structural scaffolds of proteins. Curr. Opin. Chem. Biol. 7, 12–20 10.1016/S1367-5931(02)00018-2 12547421

[26] Goldman A.D., Beatty J.T. and Landweber L.F. (2016) The TIM barrel architecture facilitated the early evolution of protein-mediated metabolism. J. Mol. Evol. 82, 17–26 10.1007/s00239-015-9722-8 26733481 PMC4709378

[27] Sterner R. and Höcker B. (2005) Catalytic versatility, stability, and evolution of the (βα)_8_-barrel enzyme fold. Chem. Rev. 105, 4038–4055 10.1021/cr030191z 16277370

[28] Rhys G.G., Wood C.W., Beesley J.L., Zaccai N.R., Burton A.J., Brady R.L. et al. (2019) Navigating the structural landscape of de novo α-helical bundles. J. Am. Chem. Soc. 141, 8787–8797 10.1021/jacs.8b13354 31066556

[29] Bermeo S., Favor A., Chang Y.-T., Norris A., Boyken S.E., Hsia Y. et al. (2022) De novo design of obligate ABC-type heterotrimeric proteins. Nat. Struct. Mol. Biol. 29, 1266–1276 10.1038/s41594-022-00879-4 36522429 PMC9758053

[30] Merljak E., Malovrh B. and Jerala R. (2023) Segmentation strategy of de novo designed four-helical bundles expands protein oligomerization modalities for cell regulation. Nat. Commun. 14, 1995 10.1038/s41467-023-37765-6 37031229 PMC10082849

[31] Rhys G.G., Cross J.A., Dawson W.M., Thompson H.F., Shanmugaratnam S., Savery N.J. et al. (2022) De novo designed peptides for cellular delivery and subcellular localisation. Nat. Chem. Biol. 18, 999–1004 10.1038/s41589-022-01076-6 35836017

[32] Cross J.A., Dawson W.M., Shukla S.R., Weijman J.F., Mantell J., Dodding M.P. et al. (2024) A de novo designed coiled coil-based switch regulates the microtubule motor kinesin-1. Nat. Chem. Biol. 20, 916–923 10.1038/s41589-024-01640-2 38849529 PMC11213707

[33] Koga N., Tatsumi-Koga R., Liu G., Xiao R., Acton T.B., Montelione G.T. et al. (2012) Principles for designing ideal protein structures. Nature 491, 222–227 10.1038/nature11600 23135467 PMC3705962

[34] Koga R. and Koga N. (2019) Consistency principle for protein design. Biophys. Physicobiol. 16, 304–309 10.2142/biophysico.16.0_304 31984185 PMC6975900

[35] Nagarajan D., Deka G. and Rao M. (2015) Design of symmetric TIM barrel proteins from first principles. BMC Biochem. 16, 18 10.1186/s12858-015-0047-4 26264284 PMC4531894

[36] Kadumuri R.V. and Vadrevu R. (2018) Diversity in αβ and βα loop connections in TIM barrel proteins: implications for stability and design of the fold. Interdiscip. Sci. Comput. Life Sci. 10, 805–812 10.1007/s12539-017-0250-7 29064074

[37] Romero-Romero S., Becerril-Sesín L.A., Costas M., Rodríguez-Romero A. and Fernández-Velasco D.A. (2018) Structure and conformational stability of the triosephosphate isomerase from Zea mays. Comparison with the chemical unfolding pathways of other eukaryotic TIMs. Arch. Biochem. Biophys. 658, 66–76 10.1016/j.abb.2018.09.022 30261166

[38] Quezada A.G., Cabrera N., Piñeiro Á., Díaz-Salazar A.J., Díaz-Mazariegos S., Romero-Romero S. et al. (2018) A strategy based on thermal flexibility to design triosephosphate isomerase proteins with increased or decreased kinetic stability. Biochem. Biophys. Res. Commun. 503, 3017–3022 10.1016/j.bbrc.2018.08.087 30143261

[39] Romero-Romero S., Costas M., Rodríguez-Romero A. and Fernández-Velasco D.A. (2015) Reversibility and two state behaviour in the thermal unfolding of oligomeric TIM barrel proteins. Phys. Chem. Chem. Phys. 17, 20699–20714 10.1039/C5CP01599E 26206330

[40] Faure A.J., Martí-Aranda A., Hidalgo-Carcedo C., Beltran A., Schmiedel J.M. and Lehner B. (2024) The genetic architecture of protein stability. Nature 634, 995–1003 10.1038/s41586-024-07966-0 39322666 PMC11499273

[41] Tsuboyama K., Dauparas J., Chen J., Laine E., Mohseni Behbahani Y., Weinstein J.J. et al. (2023) Mega-scale experimental analysis of protein folding stability in biology and design. Nature 620, 434–444 10.1038/s41586-023-06328-6 37468638 PMC10412457

[42] Romero-Romero S., Costas M., Silva Manzano D.-A., Kordes S., Rojas-Ortega E., Tapia C. et al. (2021) The stability landscape of de novo TIM barrels explored by a modular design approach. J. Mol. Biol. 433, 167153 10.1016/j.jmb.2021.167153 34271011 PMC8404036

[43] Kordes S., Romero‐Romero S., Lutz L. and Höcker B. (2022) A newly introduced salt bridge cluster improves structural and biophysical properties of *de novo* tim barrels. Protein Sci. 31, 513–527 10.1002/pro.4249 34865275 PMC8820119

[44] Koch J., Romero‐Romero S. and Höcker B. (2024) Stepwise introduction of stabilizing mutations reveals nonlinear additive effects in *de novo* tim barrels. Protein Sci. 33, e4926 10.1002/pro.4926 38380781 PMC10880431

[45] Hupfeld E., Schlee S., Wurm J.P., Rajendran C., Yehorova D., Vos E. et al. (2024) Conformational modulation of a mobile loop controls catalysis in the (βα)_8_-barrel enzyme of histidine biosynthesis HisF. JACS Au 4, 3258–3276 10.1021/jacsau.4c00558 39211614 PMC11350729

[46] Liao Q., Kulkarni Y., Sengupta U., Petrović D., Mulholland A.J., Van Der Kamp M.W. et al. (2018) Loop motion in triosephosphate isomerase is not a simple open and shut case. J. Am. Chem. Soc. 140, 15889–15903 10.1021/jacs.8b09378 30362343

[47] Romero-Rivera A., Corbella M., Parracino A., Patrick W.M. and Kamerlin S.C.L. (2022) Complex loop dynamics underpin activity, specificity, and evolvability in the (βα)_8_ barrel enzymes of histidine and tryptophan biosynthesis. JACS Au 2, 943–960 10.1021/jacsau.2c00063 35557756 PMC9088769

[48] Brown F.K. and Kollman P.A. (1987) Molecular dynamics simulations of “loop closing” in the enzyme triose phosphate isomerase. J. Mol. Biol. 198, 533–546 10.1016/0022-2836(87)90298-1 3430618

[49] Joseph D., Petsko G.A. and Karplus M. (1990) Anatomy of a conformational change: hinged “Lid” motion of the triosephosphate isomerase loop. Science 249, 1425–1428 10.1126/science.2402636 2402636

[50] Richard J.P., Zhai X. and Malabanan M.M. (2014) Reflections on the catalytic power of a TIM-barrel. Bioorganic Chem. 57, 206–212 10.1016/j.bioorg.2014.07.001 PMC4256097 25092608

[51] Richard J.P., Amyes T.L. and Reyes A.C. (2018) Orotidine 5′-monophosphate decarboxylase: probing the limits of the *possible* for enzyme catalysis. Acc. Chem. Res. 51, 960–969 10.1021/acs.accounts.8b00059 29595949 PMC6016548

[52] He R., Reyes A.C., Amyes T.L. and Richard J.P. (2018) Enzyme architecture: the role of a flexible loop in activation of glycerol-3-phosphate dehydrogenase for catalysis of hydride transfer. Biochemistry 57, 3227–3236 10.1021/acs.biochem.7b01282 29337541 PMC6001809

[53] Ray W.J., Long J.W. and Owens J.D. (1976) An analysis of the substrate-induced rate effect in the phosphoglucomutase system. Biochemistry 15, 4006–4017 10.1021/bi00663a015 963019

[54] Jian X., Sun Q., Xu W., Qu H., Feng X. and Li C. (2024) Engineering the substrate specificity of UDP‐glycosyltransferases for synthesizing triterpenoid glycosides with a linear trisaccharide as aided by ancestral sequence reconstruction. Angew. Chem. Int. Ed. 63, e202409867 10.1002/anie.202409867 39172135

[55] Marek M., Chaloupkova R., Prudnikova T., Sato Y., Rezacova P., Nagata Y. et al. (2020) Structural and catalytic effects of surface loop-helix transplantation within haloalkane dehalogenase family. Comput. Struct. Biotechnol. J. 18, 1352–1362 10.1016/j.csbj.2020.05.019 32612758 PMC7306515

[56] Heinemann P.M., Armbruster D. and Hauer B. (2021) Active-site loop variations adjust activity and selectivity of the cumene dioxygenase. Nat. Commun. 12, 1095 10.1038/s41467-021-21328-8 33597523 PMC7889853

[57] Wang T., Zhang X., Zhang O., Chen G., Pan P., Wang E. et al. (2024) Highly accurate and efficient deep learning paradigm for full-atom protein loop modeling with KarmaLoop. Research 7, 0408 10.34133/research.0408 39055686 PMC11268956

[58] Lu W., Zhang J., Huang W., Zhang Z., Jia X., Wang Z. et al. (2024) DynamicBind: predicting ligand-specific protein-ligand complex structure with a deep equivariant generative model. Nat. Commun. 15, 1071 10.1038/s41467-024-45461-2 38316797 PMC10844226

[59] Abramson J., Adler J., Dunger J., Evans R., Green T., Pritzel A. et al. (2024) Accurate structure prediction of biomolecular interactions with AlphaFold 3. Nature 630, 493–500 10.1038/s41586-024-07487-w 38718835 PMC11168924

[60] Jumper J., Evans R., Pritzel A., Green T., Figurnov M., Ronneberger O. et al. (2021) Highly accurate protein structure prediction with AlphaFold. Nature 596, 583–589 10.1038/s41586-021-03819-2 34265844 PMC8371605

[61] Lin Z., Akin H., Rao R., Hie B., Zhu Z., Lu W. et al. (2023) Evolutionary-scale prediction of atomic-level protein structure with a language model. Science 379, 1123–1130 10.1126/science.ade2574 36927031

[62] Baek M., DiMaio F., Anishchenko I., Dauparas J., Ovchinnikov S., Lee G.R. et al. (2021) Accurate prediction of protein structures and interactions using a three-track neural network. Science 373, 871–876 10.1126/science.abj8754 34282049 PMC7612213

[63] Wu R., Ding F., Wang R., Shen R., Zhang X., Luo S. et al. (2022) High-resolution *de novo* structure prediction from primary sequence.

[64] Passaro S., Corso G., Wohlwend J., Reveiz M., Thaler S., Somnath V.R. et al. (2025) Boltz-2: towards accurate and efficient binding affinity prediction.

[65] Watson J.L., Juergens D., Bennett N.R., Trippe B.L., Yim J., Eisenach H.E. et al. (2023) De novo design of protein structure and function with RFdiffusion. Nature 620, 1089–1100 37433327 10.1038/s41586-023-06415-8 PMC10468394

[66] Ingraham J.B., Baranov M., Costello Z., Barber K.W., Wang W., Ismail A. et al. (2023) Illuminating protein space with a programmable generative model. Nature 623, 1070–1078 37968394 10.1038/s41586-023-06728-8 PMC10686827

[67] Cho Y., Pacesa M., Zhang Z., Correia B.E. and Ovchinnikov S. (2025) Boltzdesign1: inverting all-atom structure prediction model for generalized biomolecular binder design.

[68] Barozet A., Chacón P. and Cortés J. (2021) Current approaches to flexible loop modeling. Curr. Res. Struct. Biol. 3, 187–191 34409304 10.1016/j.crstbi.2021.07.002 PMC8361254

[69] Kundert K. and Kortemme T. (2019) Computational design of structured loops for new protein functions. Biol. Chem. 400, 275–288 30676995 10.1515/hsz-2018-0348 PMC6530579

[70] Wang T., Wang L., Zhang X., Shen C., Zhang O., Wang J. et al. (2023) Comprehensive assessment of protein loop modeling programs on large-scale datasets: prediction accuracy and efficiency. Brief. Bioinform. 25, bbad486 38171930 10.1093/bib/bbad486 PMC10764206

[71] Jiang H., Jude K.M., Wu K., Fallas J., Ueda G., Brunette T.J. et al. (2024) De novo design of buttressed loops for sculpting protein functions. Nat. Chem. Biol. 20, 974–980 10.1038/s41589-024-01632-2 38816644 PMC11288887

[72] Anand N., Eguchi R., Mathews I.I., Perez C.P., Derry A., Altman R.B. et al. (2022) Protein sequence design with a learned potential. Nat. Commun. 13, 746 10.1038/s41467-022-28313-9 35136054 PMC8826426

[73] Goverde C.A., Pacesa M., Goldbach N., Dornfeld L.J., Balbi P.E.M., Georgeon S. et al. (2024) Computational design of soluble and functional membrane protein analogues. Nature 631,449–458 10.1038/s41586-024-07601-y 38898281 PMC11236705

[74] Chu A.E., Fernandez D., Liu J., Eguchi R.R. and Huang P.-S. (2022) De novo design of a highly stable ovoid TIM barrel: unlocking pocket shape towards functional design. BioDesign Res. 2022, 9842315 10.34133/2022/9842315 PMC10521652 37850141

[75] Ren J., Chu A.E., Jude K.M., Picton L.K., Kare A.J., Su L. et al. (2022) Interleukin-2 superkines by computational design. Proc. Natl. Acad. Sci. 119, e2117401119 10.1073/pnas.2117401119 35294290 PMC8944926

[76] Huang P.-S., Ban Y.-E.A., Richter F., Andre I., Vernon R., Schief W.R. et al. (2011) RosettaRemodel: a generalized framework for flexible backbone protein design. PloS ONE 6, e24109 10.1371/journal.pone.0024109 21909381 PMC3166072

[77] Wiese J.G., Shanmugaratnam S. and Höcker B. (2021) Extension of a *de novo* TIM barrel with a rationally designed secondary structure element. Protein Sci. 30, 982–989 10.1002/pro.4064 33723882 PMC8040861

[78] Kordes S., Beck J., Shanmugaratnam S., Flecks M. and Höcker B. (2023) Physics-based approach to extend a *de novo* TIM barrel with rationally designed helix-loop-helix motifs. Protein Eng. Des. Sel. 36, gzad012 10.1093/protein/gzad012 37707513

[79] Kandel J., Tayara H. and Chong K.T. (2021) PUResNet: prediction of protein-ligand binding sites using deep residual neural network. J. Cheminformatics 13, 65 10.1186/s13321-021-00547-7 PMC8424938 34496970

[80] Beck J., Shanmugaratnam S. and Höcker B. (2024) Diversifying *de novo* tim barrels by hallucination. Protein Sci. 33, e5001 10.1002/pro.5001 38723111 PMC11081422

[81] Anishchenko I., Pellock S.J., Chidyausiku T.M., Ramelot T.A., Ovchinnikov S., Hao J. et al. (2021) De novo protein design by deep network hallucination. Nature 600, 547–552 10.1038/s41586-021-04184-w 34853475 PMC9293396

[82] Wang J., Lisanza S., Juergens D., Tischer D., Watson J.L., Castro K.M. et al. (2022) Scaffolding protein functional sites using deep learning. Science 377, 387–394 10.1126/science.abn2100 35862514 PMC9621694

[83] Caldwell S.J., Haydon I.C., Piperidou N., Huang P.-S., Bick M.J., Sjöström H.S. et al. (2020) Tight and specific lanthanide binding in a de novo TIM barrel with a large internal cavity designed by symmetric domain fusion. Proc. Natl. Acad. Sci. 117, 30362–30369 10.1073/pnas.2008535117 33203677 PMC7720202

[84] Lin Y., Koga N., Vorobiev S.M. and Baker D. (2017) Cyclic oligomer design with de novo αβ‐proteins. Protein Sci. 26, 2187–2194 10.1002/pro.3270 28801928 PMC5654858

[85] Klein A.S., Leiss-Maier F., Mühlhofer R., Boesen B., Mustafa G., Kugler H. et al. (2024) A de novo metalloenzyme for cerium photoredox catalysis. J. Am. Chem. Soc. 146, 25976–25985 10.1021/jacs.4c04618 39115259 PMC11440500

[86] Egea P.W., Delhommel F., Mustafa G., Leiss-Maier F., Klimper L., Badmann T. et al. (2025) Inter-domain flexibility and AI-guided sequence optimization enhance de novo enzyme function.

[87] Dawson W.M., Rhys G.G. and Woolfson D.N. (2019) Towards functional de novo designed proteins. Curr. Opin. Chem. Biol. 52, 102–111 31336332 10.1016/j.cbpa.2019.06.011

[88] Beck J., Smith B.J., Zarifi N., Freund E., Chica R.A. and Höcker B. (2025) Customizing the structure of a minimal TIM barrel to craft a de novo enzyme. 10.1038/s41589-026-02250-w 42297966

[89] Lee F.S., Anderson A.G. and Olafson B.D. (2023) Benchmarking TriadAb using targets from the second antibody modeling assessment. Protein Eng. Des. Sel. 36, gzad013 37864287 10.1093/protein/gzad013

[90] Röthlisberger D., Khersonsky O., Wollacott A.M., Jiang L., DeChancie J., Betker J. et al. (2008) Kemp elimination catalysts by computational enzyme design. Nature 453, 190–195 18354394 10.1038/nature06879

[91] Listov D., Vos E., Hoffka G., Hoch S.Y., Berg A., Hamer-Rogotner S. et al. (2025) Complete computational design of high-efficiency Kemp elimination enzymes. Nature 643, 1421–1427 10.1038/s41586-025-09136-2 40533551 PMC12310539

[92] Lauko A., Pellock S.J., Sumida K.H., Anishchenko I., Juergens D., Ahern W. et al. (2025) Computational design of serine hydrolases. Scienceeadu2454 10.1126/science.adu2454 39946508 PMC12288761

[93] Braun M., Tripp A., Chakatok M., Kaltenbrunner S., Fischer C., Stoll D. et al. (2025) Computational enzyme design by catalytic motif scaffolding. Nature 649,237–245 41339546 10.1038/s41586-025-09747-9 PMC12727513

[94] Yeh A.H.-W., Norn C., Kipnis Y., Tischer D., Pellock S.J., Evans D. et al. (2023) De novo design of luciferases using deep learning. Nature 614, 774–780 10.1038/s41586-023-05696-3 36813896 PMC9946828

[95] Romero-Romero S., Braun A.E., Kossendey T., Ferruz N., Schmidt S. and Höcker B. (2024) De novo design of triosephosphate isomerases using generative language models.

[96] Vázquez Torres S., Benard Valle M., Mackessy S.P., Menzies S.K., Casewell N.R., Ahmadi S. et al. (2025) De novo designed proteins neutralize lethal snake venom toxins. Nature 639, 225–231 10.1038/s41586-024-08393-x 39814879 PMC11882462

[97] Glögl M., Krishnakumar A., Ragotte R.J., Goreshnik I., Coventry B., Bera A.K. et al. (2024) Target-conditioned diffusion generates potent TNFR superfamily antagonists and agonists. Science 386, 1154–1161 10.1126/science.adp1779 39636970 PMC12416549

[98] Sumida K.H., Núñez-Franco R., Kalvet I., Pellock S.J., Wicky B.I.M., Milles L.F. et al. (2024) Improving protein expression, stability, and function with proteinMPNN. J. Am. Chem. Soc. 146, 2054–2061 10.1021/jacs.3c10941 38194293 PMC10811672

[99] Pillai A., Idris A., Philomin A., Weidle C., Skotheim R., Leung P.J.Y. et al. (2024) De novo design of allosterically switchable protein assemblies. Nature 632, 911–920 10.1038/s41586-024-07813-2 39143214 PMC11338832

[100] Schneuing A., Harris C., Du Y., Didi K., Jamasb A., Igashov I. et al. (2024) Structure-based drug design with equivariant diffusion models. Nat. Comput. Sci. 4, 899–909 10.1038/s43588-024-00737-x 39653846 PMC11659159

[101] Pacesa M., Nickel L., Schellhaas C., Schmidt J., Pyatova E., Kissling L. et al. (2024) BindCraft: one-shot design of functional protein binders. Nature 646, 483–492 10.1038/s41586-025-09429-6 PMC12507698 40866699

[102] Dauparas J., Anishchenko I., Bennett N., Bai H., Ragotte R.J., Milles L.F. et al. (2022) Robust deep learning–based protein sequence design using ProteinMPNN. Science 378, 49–56 36108050 10.1126/science.add2187 PMC9997061

